# Comparison of clinical outcomes of anal fistula plug and endoanal advancement flap repair treating the complex anal fistula: a systematic review and meta-analysis

**DOI:** 10.1007/s13304-023-01674-6

**Published:** 2023-10-26

**Authors:** Yongkang An, Xueqing Chen, Maosheng Tian, Wenyue Qi, Jihua Gao

**Affiliations:** 1 The First Affiliated Hospital of Henan University of Chinese Medicine, Zhengzhou, China; 2 Graduate School, Hebei University of Chinese Medicine, Shijiazhuang, China; 3 Anorectal Department, The First Affiliated Hospital of Hebei University of Chinese Medicine, 389, Zhongshan East Road, Shijiazhuang, 050000 Hebei Province China; 4 Key Laboratory of Integrated Chinese Medicine and Western Medicine for Gastroenterology Research (Hebei), Shijiazhuang, China; 5 Staff Hospital of Hebei Normal University, Shijiazhuang, China

**Keywords:** Anal fistula plug, Endoanal advancement flap repair, Complex anal fistula, Meta-analysis

## Abstract

Anal fistula (AF) is a common disease with high prevalence and surgical operations are effective treatments in clinical work. There exist many well‐known surgical techniques treating complex anal fistula (CAF), however, none is ideal. To compare the superiority of Anal fistula plug (AFP) and Endoanal advancement flap repair (EAFR) for complex anal fistula. We searched worldwide databases including Pubmed, Embase, Cochrane Library, Web of Science, CNKI, WanFang, VIP, and SinoMed from their inception to March 2023. Studies comparing the outcomes of AFP and EAFR were included according to the PICO principles. The indicators of the healing rate, recurrence rate, wound infection rate, and complication rate, et al. were extracted and compared between different surgical methods. 5 RCTS and 7 non-RCTs were included in the meta-analysis with a total of 847 patients (341 patients conducted with AFP and 506 patients with EAFR). By combining the total effect of the 12 articles, we found that there was a statistical difference reporting the healing rate of AFP 48.3% and EAFR 64.4% treating the CAF (OR 0.68, 95% CI 0.30,1.55, *P* = 0.03), and EAFR has a better healing rate. However, there was no significant difference in terms of the recurrence rate (OR 1.68, 95% CI 0.80,3.54, *P* = 0.17), the wound infection rate (OR 1.82, 95% CI 0.95,3.52, *P* = 0.07), and the complication rate (OR 1.06, 95% CI 0.70,1.61, *P* = 0.77) either in the 12 articles or in the subgroup. The meta-analysis indicated that the EAFR was superior to AFP in terms of the healing rate treating the CAF, however, there were no significant differences between the two groups when it came to the recurrence rate, the wound infection rate, and the complication rate. EAFR might be one initial treatment for the complex cryptoglandular anal fistulas compared with AFP.

## Introduction

**Fig. 1:**
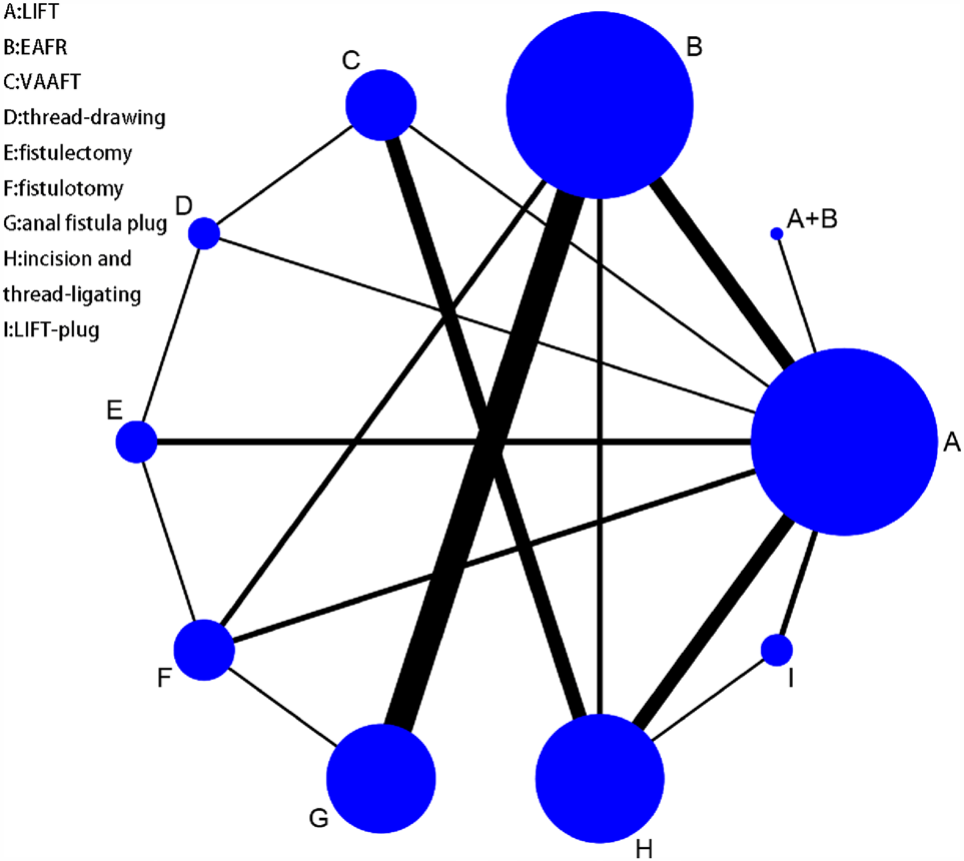
Network of the well‐known operations treating CAF

Anal fistula (AF) or a fistula-in-ano is a common disease with the prevalence is 8.6 to 10/per 100,000 of the population per year [1, 2]. The male is superior in getting the disease with a predominance of 2:1–5:1 [3]. Complex anal fistula (CAF) is one much more challenging anorectal problem with more than 30% of the external sphincter muscle being implicated [4]. There exist many well‐known techniques treating CAF——Ligation of intersphincteric fistula tract (LIFT) [5], Endoanal advancement flap repair (EAFR) [6], Video-assisted anal fistule treatment (VAAFT) [7], Thread-drawing [8], Fistulectomy [9], Fistulotomy [10], Anal fistula plug (AFP) [6], Incision and thread-ligating [11], LIFT-plug [5], et al.—leaving us unable to decide which kind of operation shall be selected. We searched the eight authoritative databases worldwide about the surgical treatments of CAF and got 3569 literature. We studied the literature and put them into a network by stataSE-64 finding numerous literature about AFP and EAFR (Fig. 1). Therefore, we studied deeply and compared the effectiveness and safety of the two kinds of surgical operations. Although there was one article [12] written by the Chinese about the comparison of AFP and EAFR in the database, there existed four serious problems: First, the article 2018 did not absorb all the pieces of literature about AFP and EAFR, and this time we tried to remedy this defect; Second, one of the literature included by the author merely had the conference abstract, which could not clarify the issue exactly. Third, no results of sensitivity analysis in that article. Fourth, the results of the qualitative assessment of Non-RCTs by the Newcastle–Ottawa Scale in that article could not be found, either. So it is urgently needed that one more precise comparison should be done between the AFP and EAFR facing those existing problems. We hope this study will increase surgeons’ understanding of the CAF and contribute to clinical decision-making and research.

Methods.

### Search strategy

We searched worldwide databases including Pubmed, Embase, Cochrane Library, Web of Science, CNKI, WanFang, VIP, and SinoMed from their inception to March 2023. The research strategy is given below.

### Researching strategy

#1 Anal Fistula[MeSH Terms] OR (anal fistula[Title/Abstract]) OR fistula-in-ano[Title/Abstract] OR (complex anal fistula[Title/Abstract]).

#2 (Endoanal advancement flap[Title/Abstract])) OR (EAFR[Title/Abstract]) OR (endorectal advancement flap[Title/Abstract]) OR (EFAR[Title/Abstract]) OR (advancement flap[Title/Abstract]) OR (Mucosal Advancement Flap[Title/Abstract]) OR (Anal fistula plug[Title/Abstract]) OR (fistula plug[Title/Abstract]) OR (AFP[Title/Abstract]).

#3 #1 AND #2

### Inclusion criteria

Two researchers independently included the literature that met the criteria according to the PICO principles [13, 14]. Targeted literature for inclusion: (1) P: Inclusion criteria: patients with a definitive diagnosis of complex anal fistula: fistula tract crossing more than 30% of the external sphincter, anterior fistula in a woman, multiple tracts, recurrent fistula, or pre-existing incontinence; Exclusion criteria: patients with other causes of anal fistula such as inflammatory bowel disease, tuberculosis, trauma, and foreign body infection; patients with other underlying diseases at the time of treatment, such as severe cardiovascular disease, diabetes mellitus, hematologic disease, and psychiatric disease were excluded; (2) I: Anal fistula plug (AFP); (3) C: Endoanal Advancement Flap (EAFR); (4) O: with definite anal fistula cure results. In case of disagreement between the two investigators, a consensus result was obtained after consultation and discussion and consultation with the supervisor.

We get 12 studies finally and their characteristics are clarified below (Table 1).

**Table 1:** Characteristics of included studies

No.	Research	Country	Research type	Operation	Anal fistula type
1	Abbas (2011) [15]	America	Non-RCT	AFP EAFR	Complex cryptoglandular fistula
2	Adamina M2010 [3]	Canada	Non-RCT	AFP EAFR	High trans-sphincteric cryptoglandular anal fistula
3	Bondi (2017) [6]	Norway Sweden	RCT	AFP EAFR	Trans-sphincteric complex anal fistula
4	Fisher OM2015 [16]	Switzerland	Non-RCT	AFP EAFR	Anal fistula more than one-third of the external anal sphincter
5	Ortiz (2009) [17]	Spain	RCT	AFP EAFR	High trans-sphincteric cryptoglandular fistula more than two-thirds of the external sphincter complex
6	Schwandner (2018) [5]	Germany	RCT	AFP EAFR	Transsphincteric anal fistulas
7	Van Koperen(2011) [18]	Netherlands	RCT	AFP EAFR	High transsphincteri cryptoglandular fistula
8	Wang JY2009 [19]	America	Non-RCT	AFP EAFR	High trans-sphincteric cryptoglandular anal fistula
9	Ba-bai-ke-re (2010) [20]	China	RCT	AFP EAFR	Complex cryptoglandular fistula
10	Christof (2009) [21]	America	Non-RCT	AFP EAFR	High trans-sphincteric cryptoglandular anal fistula
11	Chung (2009) [22]	America	Non-RCT	AFP EAFR	High trans-sphincteric fistulas of cryptoglandular origin
12	Ellis (2007) [23]	America	Non-RCT	AFP EAFR	Complex cryptoglandular fistula

No.	Mean age (range)	Sample size	Male/Female	Follow up (Months)	Outcome indicator
1	45 (22–73) 45 (22–73)	31	12/19	2–40	Effective or recurrence rate;infection rate;complication
2	47.2 47.3	24	12/12	28.1 (7.4–43.9) 14.1 (2–160.7)	Effective or recurrence rate;hospital days and complication
3	42.2 (25.7–65.3) 53.1 (22.0–69.8)	94	48/46	12 (9–24) 12 (9–24)	Effective or recurrence rate;infection rate;complication
4	41 (34–51) 44 (34–58)	71	31/40	6 (2–12) 6 (2–12)	Effective or recurrence rate;infection rate;complication
5	46.5 (30–76) 46.5 (30–76)	32	16/16	12	Effective or recurrence rate;infection rate;complication
6	45.1 ± 13.1 49.5 ± 13.2	92	43/39	12	Effective or recurrence rate;duration of operation;infection rate;complication
7	45 (24–79) 42 (24–61)	42	23/19	11 (5–27)	Effective or recurrence rate;duration of operation;wexnerscore
8	40 (19–67) 39 (29–58)	55	29/26	9.3 (3.7–23) 27.3 (3.1–64.3)	Healing rate
9	44.8 (18–59) 45.1 (17–61)	90	45/45	5.7 (5.1–6.4) 6.1 (5.9–6.5)	Effective or recurrence rate;duration of pain;healing time; complication
10	48.3 ± 12.0 47 ± 11.5	73	30/43	14 (6–22) 56 (6–136)	Effective or recurrence rate; complication
11	46 (23–68) 46 (28–75)	123	27/96	6	HHealing rate and wound infection rate
12	32 (21–56) 42 (21–69)	113	18/95	10 (6–22) 6 (3–11)	Effective or recurrence rate; VAS score

### Data extraction and quality assessment

Two investigators independently extracted the literature including author names, published year, journal name, country, and where the study has been done [24]. The information of the article was extracted such as age, sex, fistula type, operation type, postoperative pain, VAS score, Wexner score, healing rate, recurrence rate, wound infection rate, complication rate, BMI, duration of operation, cost, hospital stay, study design, comparison, follow-up, et. al.

Quality assessment of the study was done scrupulously by researchers. The quality of RCTs was assessed by the Cochrane Handbook for Systematic Reviews in the Review Manage [25], while the Non-RCTs’ quality was assessed by the Newcastle–Ottawa Scale [13, 26].

### Statistical analysis

The Review Manage software version 5.4 was used for the meta-analysis. The healing rate, recurrence rate, wound infection rate, and complication rate were compared between AFP and EAFR by the OR and 95% CI. *I*^2^ was used to evaluate the heterogeneity of the included studies. It was considered low heterogeneity if *I*^2^ < 50% or *P* value more than 0.05 for *Q* statistical choosing the fixed effect model. Otherwise, the random fixed effect model was selected when *I*^2^ ≧ 50% or the *P* value was less than 0.05 [27, 28].

### Sensitivity analysis of studies

The sensitivity analysis was done by excluding one study sequentially and the investigators wrote down the 95% CI, *P* value, and *I*^2^, which were recalculated by the remaining studies, to assess whether the excluding study affected the result markedly with a small sample size [26].

### Assessment of risk of bias

The quality of RCTs was assessed by the Cochrane Handbook for Systematic Reviews in the Review Manage, while the Non-RCTs’ quality was assessed by the Newcastle–Ottawa Scale.

## Result

### Search results and study characteristics

1995 literature had been obtained after searching the eight authoritative databases worldwide. There were 1439 articles remaining after duplicating the 556 overlapped literature finally. Then 1377 records were excluded with the reasons of low simple anal fistula, meta-analysis, protocol, review, graduation thesis, case report, animal experiment, children, conference summary, tumor or tuberculosis, Crohn’s disease, and letter, leaving 62 articles. According to the PICO principles, 12 studies were included meeting the inclusion criteria.

**Fig. 2:**
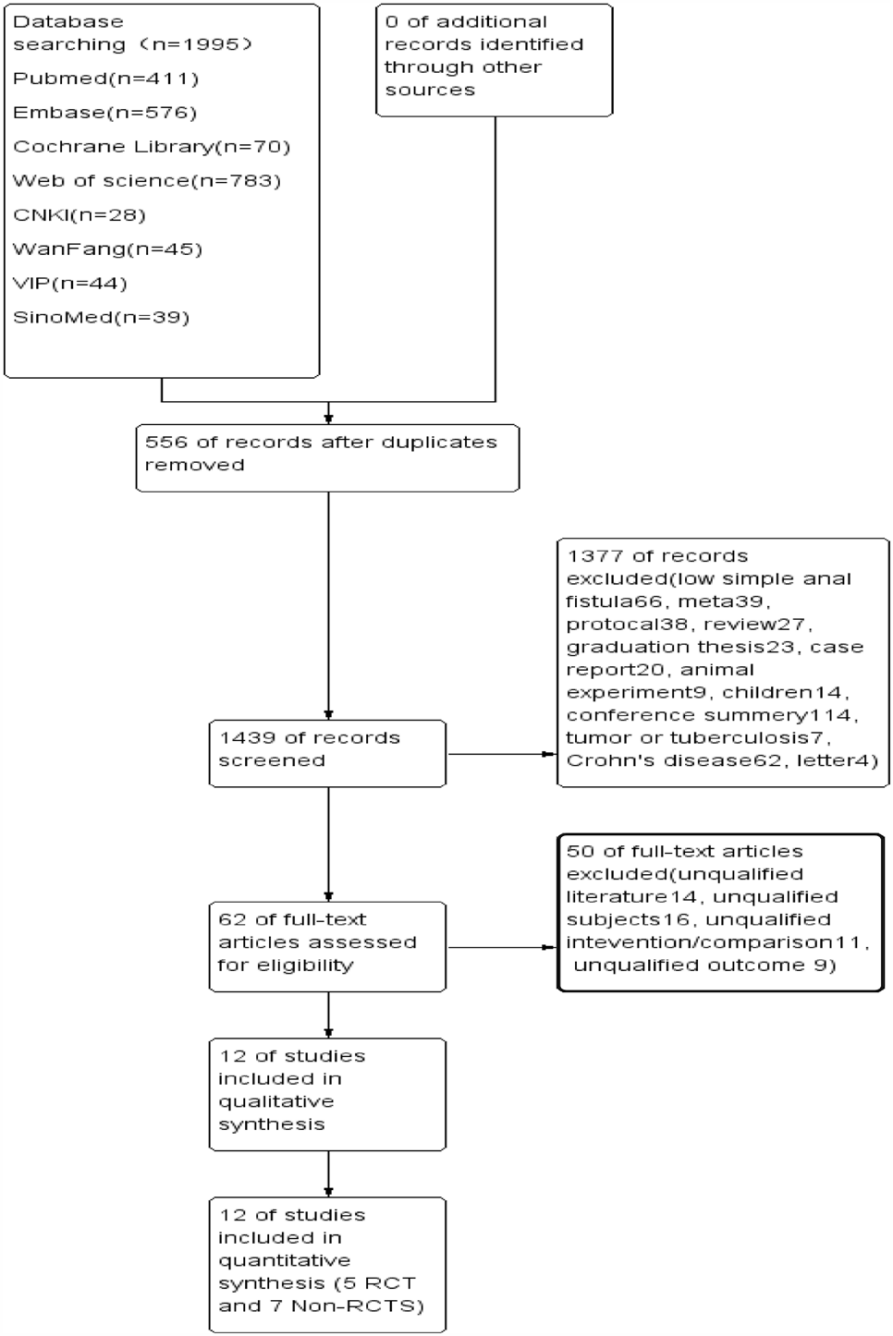
Flowchart of study selection

5 RCTS and 7 non-RCTs were included in our meta-analysis. The detailed flow diagram is shown in Fig. 2. A total of 847 patients were included in our analysis with 341 patients being conducted with AFP and 506 patients with EAFR. The study characteristics are shown in Table 1.

### Healing rate

**Fig. 3:**
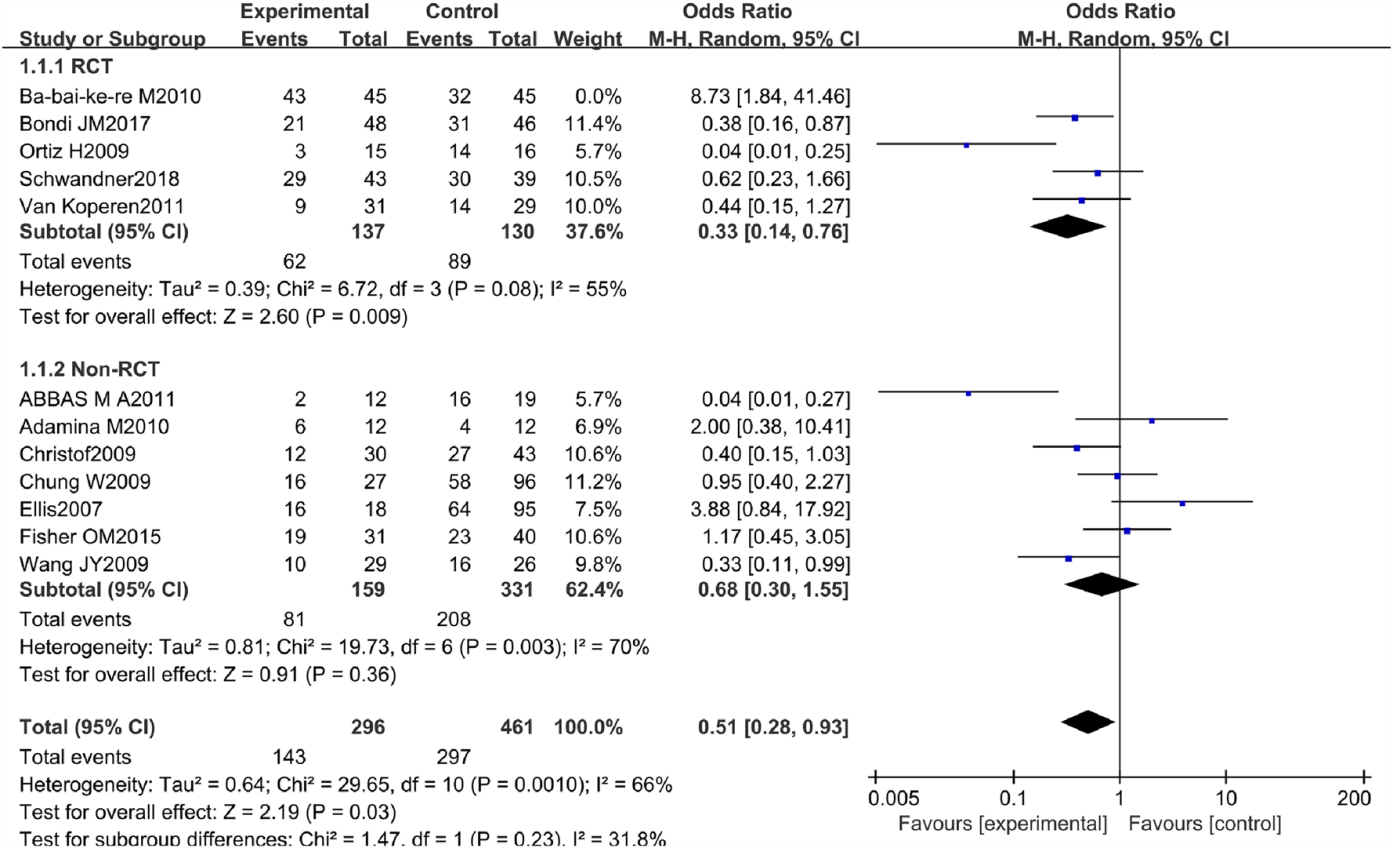
Forest plot of the comparison for healing rate

There were 5 RCTs and 7 non-RCTs reporting the healing rate of AFP 48.3% and EAFR 64.4% treating the CAF. We combined their effect and got an outcome (Fig. 3). However high heterogeneity was found in the combined effect, so the sensitivity analysis was done by excluding one study sequentially, and the remaining studies were recalculated at last. The study of Ba-bai-ke-re (2010) was excluded because it affected the combined result markedly with a small sample size. However, the result of heterogeneity was still high. The reasons for the high heterogeneity may be that some patients came from different countries or centers and the follow-up time was not long enough. Finally, we chose the random effect model to pool the ORs because of the existing real heterogeneity. The details of the excluded studies are shown in Table 2.

**Table 2:** Sensitive analysis of the included studies in terms of healing rate

Sensitivity analysis of the healing rate				
	Excluding study	MD [95% CI]	*P* value	*I*^2^ (%)
1	Abbas (2011)	0.73 [0.39, 1.37]	0.33	70
2	Adamina (2010)	0.57 [0.28, 1.12]	0.1	75
3	Ba-bai-ke-re (2010)	0.51 [0.28, 0.93]	0.03	66
4	Bondi (2017)	0.65 [0.31, 1.35]	0.25	75
5	Christof (2009)	0.64 [0.31, 1.33]	0.24	75
6	Chung (2009)	0.59 [0.28, 1.22]	0.15	75
7	Ellis (2007)	0.54 [0.28, 1.03]	0.06	72
8	Fisher (2015)	0.57 [0.28, 1.18]	0.13	75
9	Ortiz (2009)	0.73 [0.39, 1.37]	0.33	70
10	Schwandner (2013)	0.61 [0.29, 1.28]	0.19	76
11	Van Koperen (2011)	0.64 [0.31, 1.31]	0.22	76
12	Wang (2009)	0.65 [0.32, 1.34]	0.24	75

**Fig. 4:**
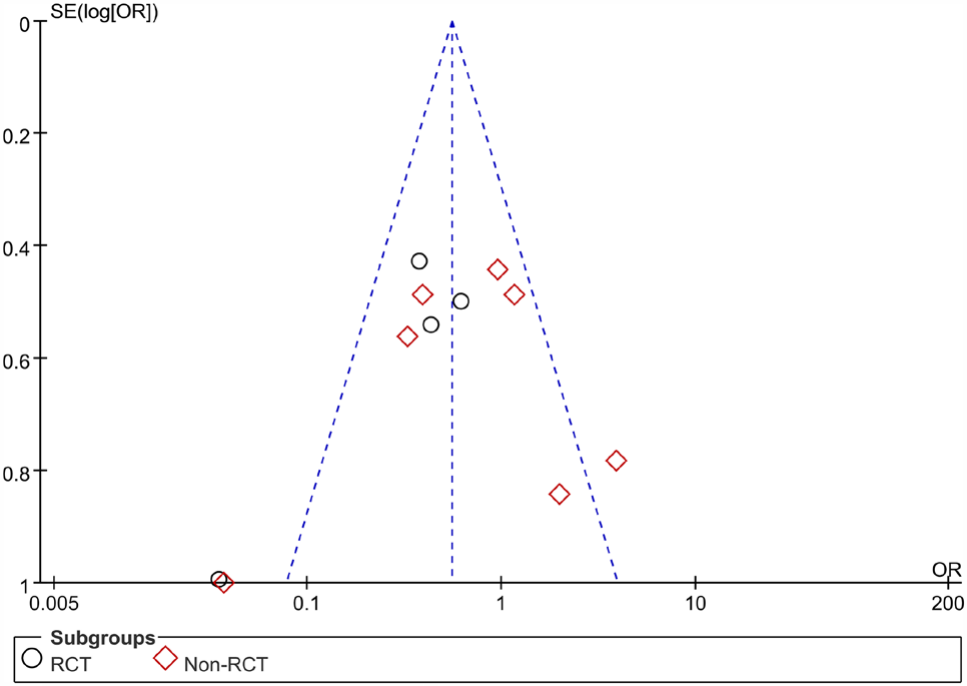
Funnel plot of the comparison for the healing rate in terms of publication bias

The pooled result of the RCTs (OR 0.33, 95% CI 0.14, 0.76, *P* = 0.009) and non-RCTs (OR 0.68, 95% CI 0.30, 1.55, *P* = 0.36) revealed that there was a statistical difference in terms of healing rate between the AFP and EAFR groups (*P* = 0.03), and EAFR has a better healing rate (Fig. 4).

### Recurrence rate

**Fig. 5:**
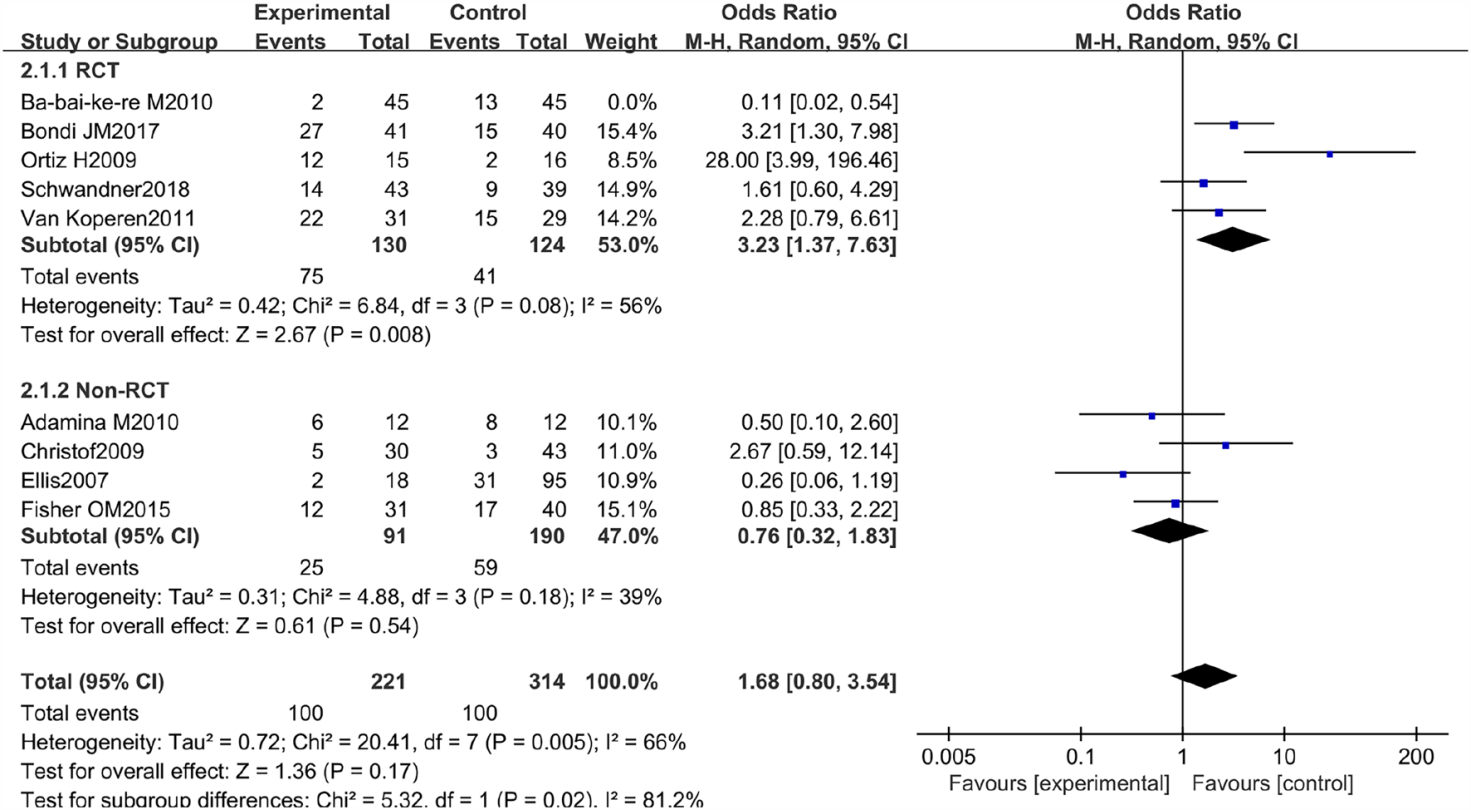
Forest plot of the comparison for recurrence rate

There were 5 RCTs and 4 non-RCTs reporting the recurrence rate of AFP 45.2% and EAFR 31.8% treating the CAF. We combined their effect and got an outcome (Fig. 5). However high heterogeneity was found in the combined effect, so a sensitivity analysis was also done. The study of Ba-bai-ke-re (2010) was excluded again because it similarly affected the combined result markedly with a small sample size. At last, the result of heterogeneity was still high. The reasons for this high heterogeneity may be that patients came from different countries or that the follow-up time of some patients was not long enough. In the same way, we chose the random effect model to pool the ORs. The details of the excluded studies are shown in Table 3.

**Table 3:** Sensitive analysis of the included studies in terms of recurrence rate

Sensitivity analysis of the recurrence rate				
	Excluding study	MD [95%CI]	*P* value	*I*^2^ (%)
1	Adamina (2010)	1.43 [0.59, 3.48]	0.43	76
2	Ba-bai-ke-re (2010)	1.68 [0.80, 3.54]	0.17	66
3	Bondi (2017)	1.12 [0.45, 2.82]	0.80	74
4	Christof (2009)	1.18 [0.48, 2.94]	0.71	77
5	Ellis (2007)	1.55 [0.67, 3.59]	0.31	73
6	Fisher (2015)	1.37 [0.52, 3.58]	0.52	77
7	Ortiz (2009)	1.00 [0.47, 2.12]	1.00	68
8	Schwandner (2013)	1.25 [0.47, 3.31]	0.66	78
9	Van Koperen (2011)	1.19 [0.46, 3.07]	0.72	77

**Fig. 6:**
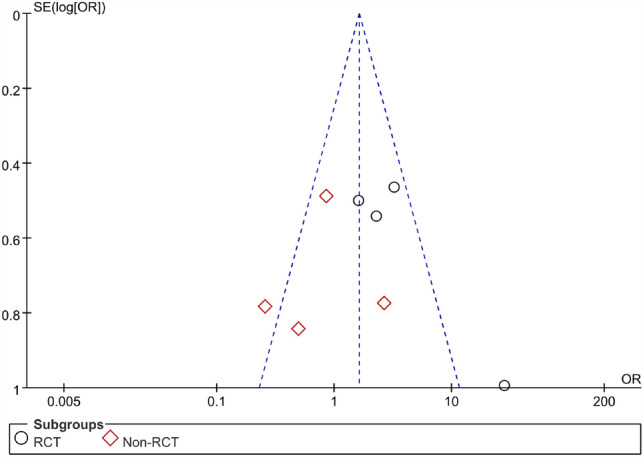
Funnel plot of the comparison for the recurrence rate in terms of publication bias

The pooled result of the RCTs (OR 3.23, 95% CI 1.37, 7.63, *P* = 0.008) and non-RCTs (OR 0.76, 95% CI 0.32, 1.83, *P* = 0.54) revealed that there was no significant difference in terms of recurrence rate between the AFP and RAF groups (OR 1.68, 95% CI 0.80, 3.54, *P* = 0.17) (Fig. 6).

### Wound infection rate

**Fig. 7:**
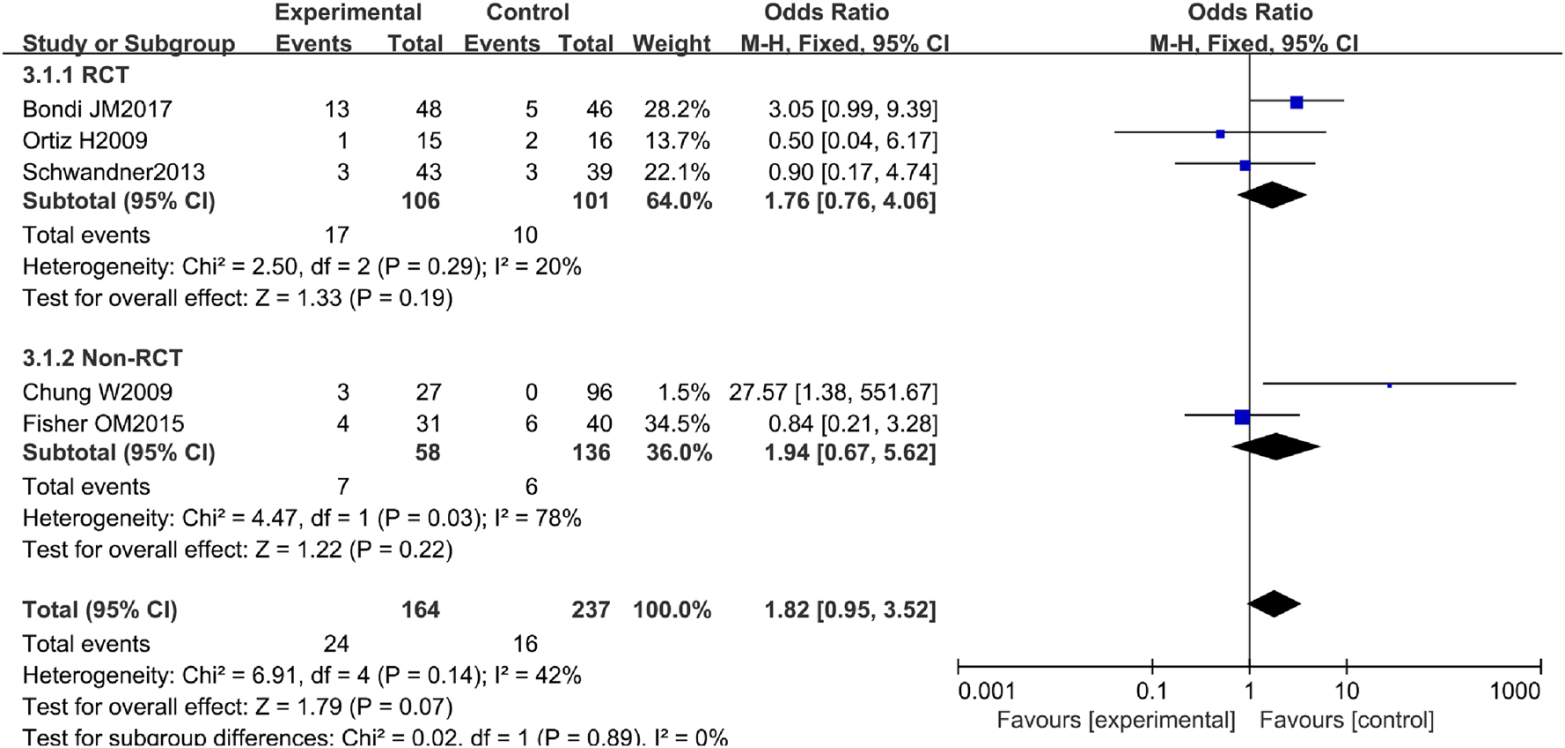
Forest plot of the comparison for wound infection rate

The wound infection rate, which included infections in the operation area after the surgery, was defined as surgical wound infection developing after the surgical treatment. There were 3 RCTs and 2 non-RCTs reporting the wound infection rate of AFP 14.6% and EAFR 6.8% treating the CAF. We combined their effect and got an outcome (Fig. 7). And the heterogeneity was not high in the combined effect with *I*^2^ = 42%. Therefore, we chose the fixed effect model to pool the ORs.

**Fig. 8:**
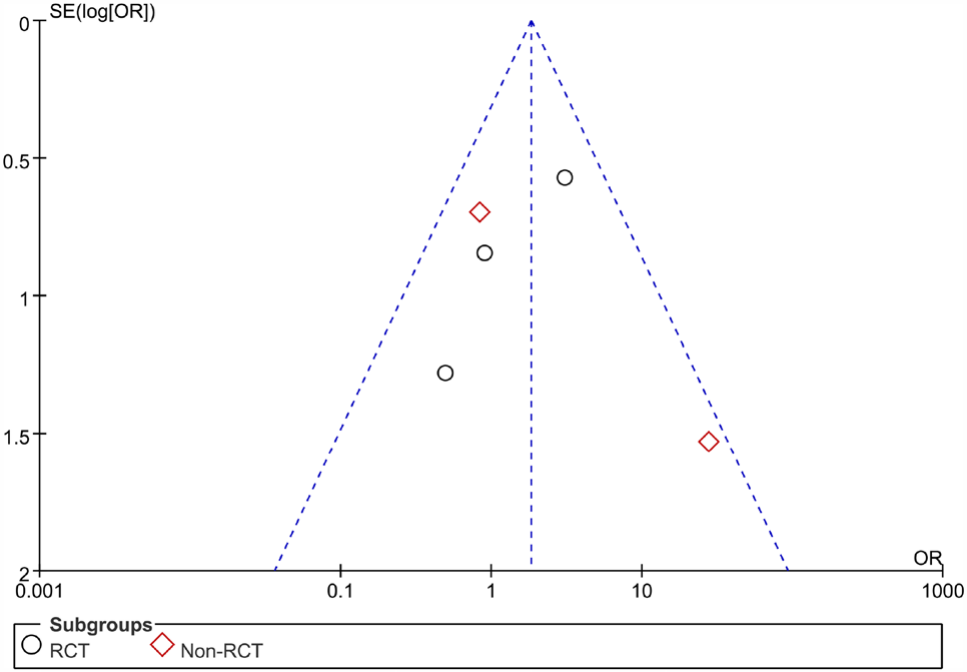
Funnel plot of the comparison for the wound infection rate in terms of publication bias

The pooled result of the RCTs (OR 1.76, 95% CI 0.76, 4.06, *P* = 0.19) and non-RCTs (OR 1.94, 95% CI 0.67, 5.62, *P* = 0.22) revealed that there was no significant difference in terms of the wound infection rate between the AFP and RAF groups (OR 1.82, 95% CI 0.95, 3.52, *P* = 0.07) (Fig. 8).

### Complication rate

**Fig. 9:**
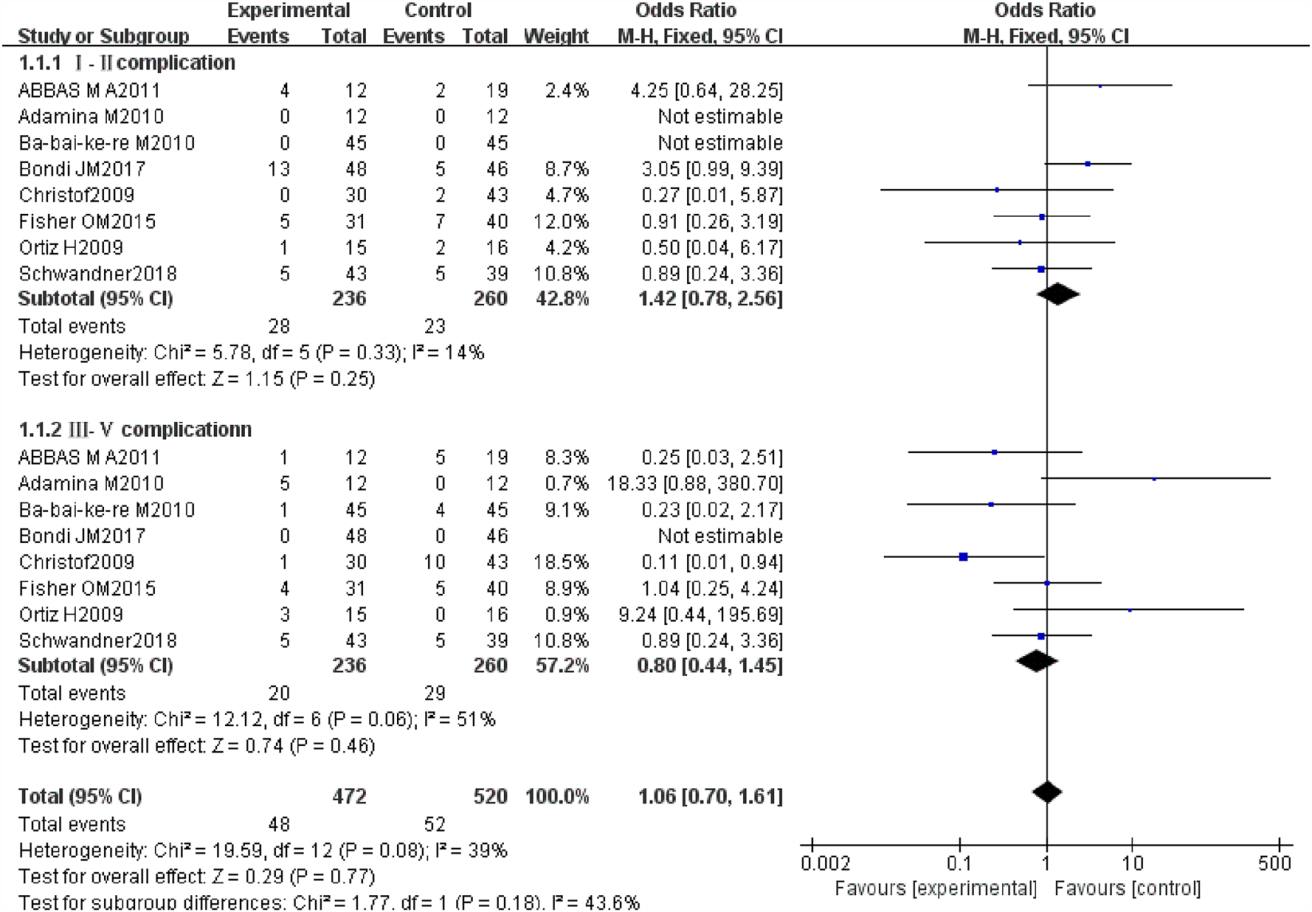
Forest plot of the comparison for complication rate

Complications refer to the occurrence of another disease or symptom during the treatment, including postoperative urinary retention, infection, postoperative bleeding, wound fissure, or anal incontinence. The Dindo–Clavien [29] classification of surgical procedures was applied to better value minor and major complications of the two techniques. There were 4 RCTs and 4 non-RCTs reporting the complication rate after the surgery. We combined their effect and got an outcome (Fig. 9). The heterogeneity could be accepted to some extent in the combined effect with *I*^2^ = 39%. And we chose the fixed effect model to pool the ORs.

The rates of gradeI–II complications for AFP and EAFR were 11.9% and 8.8%, but the difference was not statistically significant (OR 1.42, 95% CI 0.78, 2.56, *P* = 0.25). The rates of the grade III–V complications for AFP and EAFR were 8.5% and 11.2%, however, the difference was equally not statistically significant (OR 0.80, 95% CI 0.44, 1.45, *P* = 0.46). The pooled result of the grade I–II complications and the grade III–V complications for AFP and EAFR revealed that there was no significant difference (OR 1.06, 95% CI 0.70,1.61, *P* = 0.77).

**Fig. 10:**
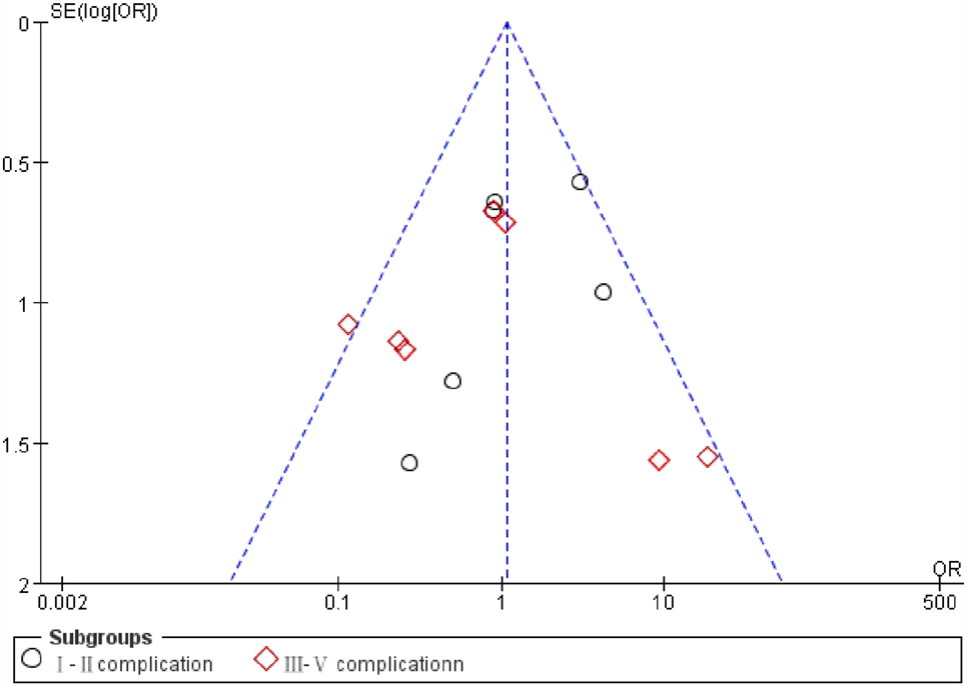
Funnel plot of the comparison for the complication rate in terms of publication bias

When it comes to the incontinence rate, there were 2 RCTs (Ba-bai-ke-re M 2010 and Christof 2009) and 1 Non-RCT (Abbas 2011) reporting the incontinence rate of AFP and EAFR treating the CAF. We did not combine their effect considering the number of 3 articles was too small. The article A-bai-ke-re 2010 revealed that there was no statistical difference between the AFP and EAFR groups (2.2% VS 8.9%) in terms of incontinence rate (*P* > 0.05) (Fig. 10).

### Duration of operation

There were three RCTs (Adamina 2010, Schwandner 2018, and Van Koperen 2011) reporting the duration of operation of AFP and EAFR treating the CAF. However, We were not able to combine their effect because only one article’s data gave the standard deviation, and the left two articles merely gave the range of duration of the operation. Otherwise, The article by Adamina (2010) indicated that the mean operation time (min. range) of the AFP (20, 10–50) and EAFR (60, 45–105) revealed there was a statistical difference between the two groups (*P* = 0.0005).

### Hospital days

There were three non-RCTs (Adamina 2010, Fisher 2015, and Wang 2009) reporting the hospital days of AFP and EAFR treating the CAF. However, We were not able to combine their effect because these articles’ data only gave the standard deviation or merely the range of duration of the operation. Otherwise, the article by Adamina M. 2010 indicated that there was a statistical difference between the AFP and EAFR groups with a median stay of 1 day versus 2.5 days (*P* = 0.0002).

### Wexner score

There were 2 RCTs (Bondi 2017 and Van Koperen 2011) reporting the Wexner score of AFP and EAFR treating the CAF. We were not able to combine their effect because these articles’ data only gave the standard deviation or merely the range of the Wexner score. The article by Bondi (2017) revealed that there was no statistical difference between the AFP and EAFR groups in terms of the Wexner score.

### VAS score

Only one Non-RCT (Ellis 2007) reported the VAS score of AFP and EAFR treating the CAF. The article revealed that there was no statistical difference between the AFP and EAFR groups in terms of VAS scores.

### Cost

There were 2 Non-RCTs (Adamina 2010 and Fisher 2015) reporting the cost of AFP and EAFR treating the CAF. However, We were not able to combine their effect considering the number of two articles was too small. However, the article by Fisher (2015) indicated that there was a statistical difference between the AFP and EAFR groups with a median saving of $1588 in the AFP group (*P* = 0.021).

### Subgroup meta-analysis of the studies in terms of long enough follow-up

Considering the high heterogeneity found in the combined effect in terms of the healing rate and recurrence rate with the possible reason of not long enough follow-up, we decided to perform a subgroup analysis in terms of a long enough follow-up of more than 1 year. There were 3 RCTs (Bondi 2017, Ortiz 2009, and Schwandner 2018) and 2 Non-RCTs (Adamina 2010 and Christof 2009) meeting the inclusion criteria.

**Fig. 11:**
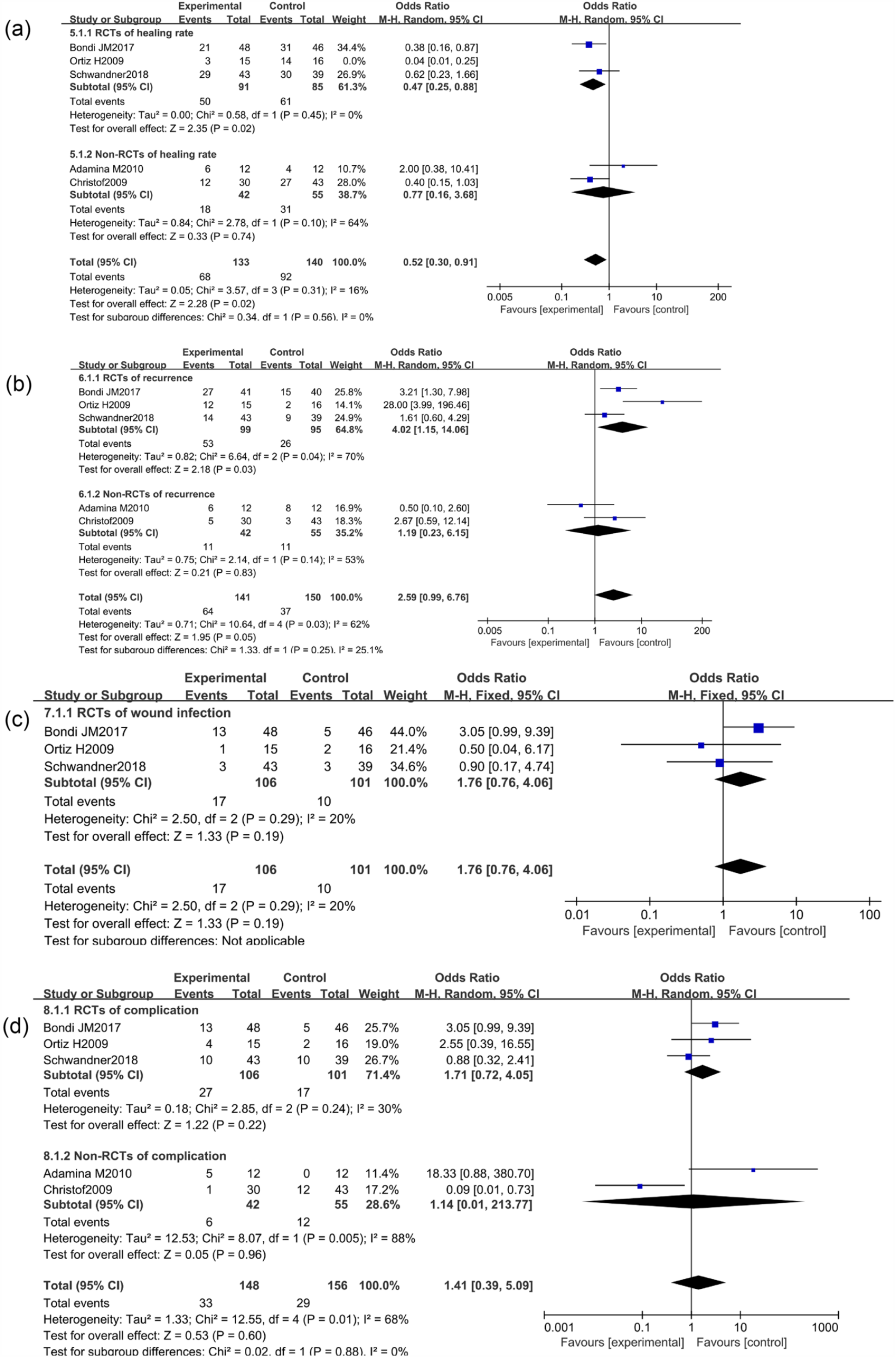
Subgroup analysis of the studies in terms of long enough follow-up

The pooled result of the RCTs (OR 0.27, 95% CI 0.08, 0.92, *P* = 0.04) and non-RCTs (OR 0.77, 95% CI 0.16, 3.68, *P* = 0.74) revealed that there was a statistical difference in terms of healing rate between the AFP 51.1% and EAFR 65.7% groups (*P* = 0.04 *I*^2^ = 61%), and EAFR has a better healing rate. Considering the heterogeneity of the included studies, the article Ortiz H2009 was excluded because of its markedly affecting the result with *I*^2^ = 61%. However, there was still a statistical difference in terms of healing rate between the two groups after the article Ortiz H2009 was excluded with *P* = 0.02 and *I*^2^ = 16%. Otherwise, there was no significant difference in terms of the recurrence rate (AFP 45.4% VS EAFR 24.7%), wound infection rate (AFP 16.0% VS EAFR 9.9%), and complication rate (AFP 22.3% VS EAFR 18.6%) with *P* = 0.05, *P* = 0.19, and *P* = 0.96 (Fig. 11).

### Assessment of risk of bias

**Fig. 12:**
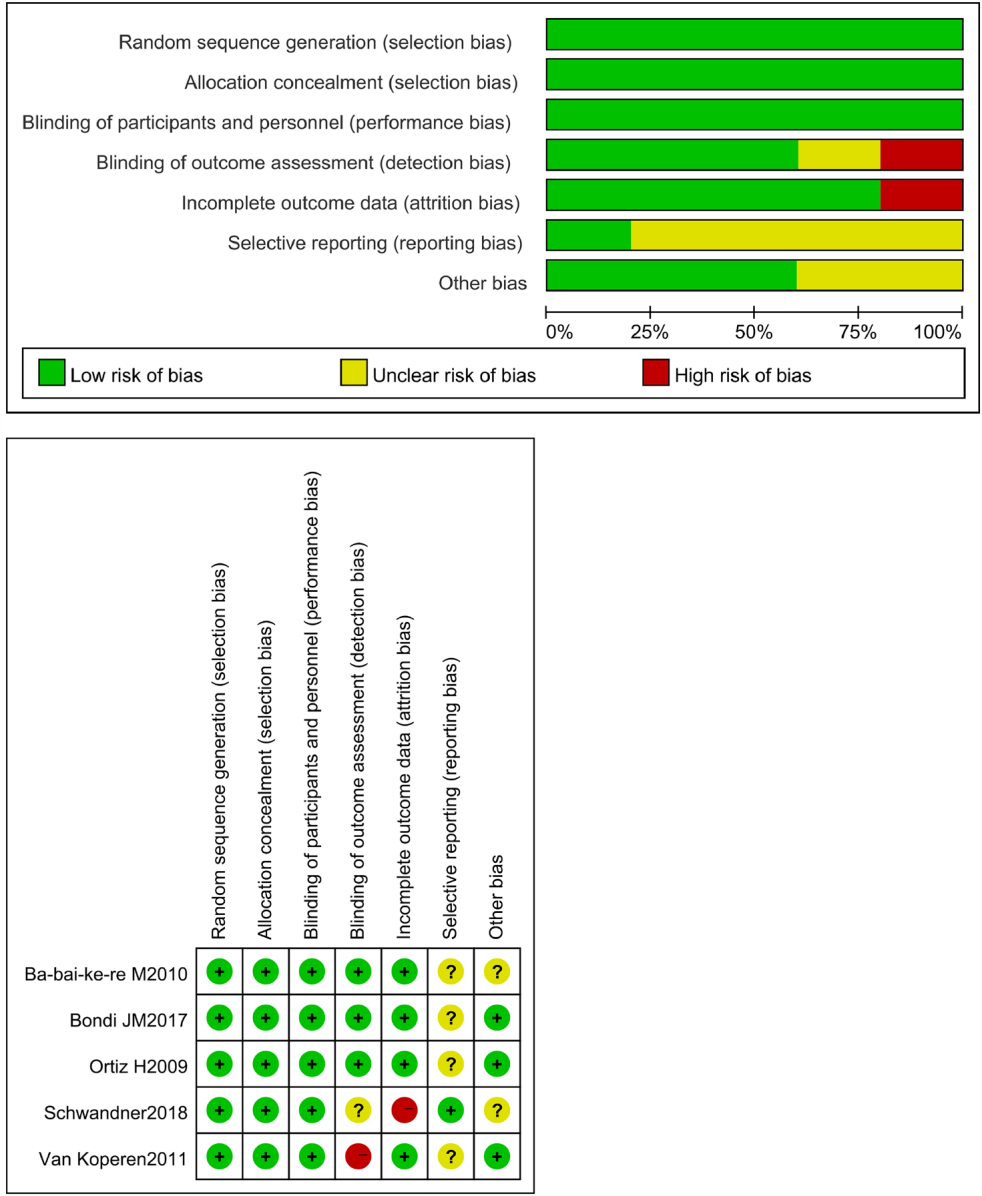
Assessment of risk of bias of RCTS

The quality of 5 RCTs was assessed by the Cochrane Handbook for Systematic Reviews and the 7 Non-RCTs’ quality was assessed by the Newcastle–Ottawa Scale. The details and results are shown in Fig. 12 and Table 4.

**Table 4:** Assessment of risk of bias of Non-RCTS

Quality evaluation of the eligible studies with Newcastle–Ottawa scale									
Study	Representativeness	Selection of non-exposed	Ascertainment of exposure	Outcome not present at the start	Comparability of most important factors	Comparability on other risk factors	Assessment of outcome	Long enough follow-up (median ≥ 1 year)	Adequacy (completeness of follow-up)
Abbas et al	**✬**	**✬**	**✬**	**✬**	**✬**	0	**✬**	0	**✬**
Adamina et al	**✬**	**✬**	**✬**	**✬**	**✬**	0	**✬**	0	**✬**
Christof et al	**✬**	**✬**	**✬**	**✬**	**✬**	**✬**	**✬**	**✬**	**✬**
Chung et al	**✬**	**✬**	**✬**	**✬**	**✬**	0	0	0	**✬**
Ellis et al	**✬**	**✬**	**✬**	**✬**	**✬**	0	0	0	**✬**
Fisher et al	**✬**	**✬**	**✬**	**✬**	**✬**	0	**✬**	0	**✬**
Wang et al	**✬**	**✬**	**✬**	0	**✬**	0	0	**✬**	**✬**
Abbas et al	**✬**	**✬**	**✬**	**✬**	**✬**	0	**✬**	0	**✬**

## Discussion

The treatment of a complex anal fistula is a hard nut to crack [30]. Although there are many kinds of surgical techniques in the medical field such as LIFT, EAFR, VAAFT, Thread-drawing, Fistulectomy, Fistulotomy, AFP, Incision and thread-ligating, LIFT-plug et al., it can be reasoned out that none of the operations is ideal [31]. However, we have to choose one better technique that meets the patients’ needs. We put all the literature about the operation of CAF into a network and found that the AFP and EAFR were widely used by surgeons. The anal fistula plug was inserted through the internal opening and pulled through the external opening until it fits snugly. The external opening was left open to allow drainage. According to Adamina, a broad-based rhomboid flap of the mucosa, submucosa, and a small portion of the internal sphincter was then raised with electrocautery until it covered the internal opening without any tension. The internal opening was closed and the external opening was left open to allow drainage [3]. To analyze which one is superior in the two kinds of operation, the comparison of AFP and EAFR is performed by meta-analysis.

There were five RCTS and seven non-RCTS included in our meta-analysis with a total of 847 patients. We studied the 12 articles with their data extracted including age, sex, fistula type, operation type, postoperative pain, VAS score, Wexner score, healing rate, recurrence rate, wound infection rate, complication rate, BMI, duration of operation, cost, hospital stay, study design, comparison, follow-up, et al. and got some meaning results.

By combining the total effect of the 12 articles, we found that there was a statistical difference reporting the healing rate of AFP 48.3% and EAFR 64.4% treating the CAF (OR 0.68, 95% CI 0.30, 1.55, *P* = 0.03), and EAFR has a better healing rate. Considering the high heterogeneity found in the combined effect with the possible reason for follow-up, we performed a subgroup analysis in terms of a long enough follow-up of more than 1 year. The pooled result of the RCTs (OR 0.47, 95% CI 0.25, 0.88, *P* = 0.02) and non-RCTs (OR 0.77, 95% CI 0.16, 3.68, *P* = 0.74) revealed that there was still a statistical difference in terms of healing rate between the AFP 51.1% and EAFR 65.7% groups (*P* = 0.02 *I*^2^ = 16%), and EAFR has a better healing rate. Otherwise, there was no significant difference in terms of the recurrence rate, the wound infection rate, and the complication rate either in the 12 articles or in the subgroup.

When it came to the comparison of the duration of operation, the hospital days, the Wexner score, the incontinence rate, the VAS score, and the cost between the AFP and EAFR group, we were not able to combine their effect because the articles related were too small. However, some worthy results could be absorbed from some of the 12 original articles—the AFP is superior in terms of the duration of operation, the hospital days, and the cost.

Some limitations were related to our study to some extent that may affect the real effect. Firstly, we only got five RCTs in the analysis which was too small to illustrate the issue. Secondly, high heterogeneity existed in the studies with the possible reasons of different countries, different surgical techniques, or follow-up. Thirdly, the hospital stay and duration of operation are both very long for a rectal flap for an anal fistula, this could be an aspect that influences the results of the study. All of the problems shall be solved by more prospective random clinical trials in the future.

In summary, the meta-analysis indicated that the EAFR was superior to AFP in terms of the healing rate treating the CAF, however, there were no significant differences between the two groups when it came to the recurrence rate, the wound infection rate, and the complication rate. EAFR might be one initial treatment for the complex cryptoglandular anal fistulas compared with AFP.

## Data Availability

The following data are publicly available. Further inquiries can be directed to the corresponding author, including the template data collection forms, data extracted from included studies, data used for all analyses, the analytic code, and any other materials used in the review.

## Declarations

### Guarantor

Jihua Gao.

### Provenance and peer review

Not commissioned, externally peer-reviewed.
